# *NAA10*-related syndrome

**DOI:** 10.1038/s12276-018-0098-x

**Published:** 2018-07-27

**Authors:** Yiyang Wu, Gholson J. Lyon

**Affiliations:** Stanley Institute for Cognitive Genomics, Cold Spring Harbor Laboratory, Woodbury, NY 11797 USA

**Keywords:** Protein folding, Protein folding

## Abstract

*NAA10*-related syndrome is an X-linked condition with a broad spectrum of findings ranging from a severe phenotype in males with p.Ser37Pro in NAA10, originally described as Ogden syndrome, to the milder *NAA10*-related intellectual disability found with different variants in both males and females. Although developmental impairments/intellectual disability may be the presenting feature (and in some cases the only finding), many individuals have additional cardiovascular, growth, and dysmorphic findings that vary in type and severity. Therefore, this set of disorders has substantial phenotypic variability and, as such, should be referred to more broadly as *NAA10*-related syndrome. *NAA10* encodes an enzyme NAA10 that is certainly involved in the amino-terminal acetylation of proteins, alongside other proposed functions for this same protein. The mechanistic basis for how variants in *NAA10* lead to the various phenotypes in humans is an active area of investigation, some of which will be reviewed herein.

## Developmental disorders: Finding mutations associated with a rare syndrome

A detailed overview of a rare X-linked hereditary disorder gives clinicians a resource for making an informed diagnosis based on genetic data and developmental abnormalities. Around 80% of all human proteins are modified on their amino terminus via tagging with an acetyl group, and the NAA10 enzyme plays a major role in this process. Mutations in the gene encoding NAA10 produce severe neurological and cardiovascular effects. Yiyang Wu and Gholson Lyon at the Cold Spring Harbor Laboratory, Woodbury, USA, have reviewed current research to facilitate accurate identification of ‘*NAA10*-related syndrome’. Since this gene resides on the X chromosome, mutations strongly affect males, although some female carriers also show symptoms. *NAA10*-related syndrome is exceedingly rare, with only 26 cases reported to date, and the researchers describe both known causative mutations and unrelated disorders that produce similar developmental defects.

## Introduction

Protein acetylation occurs both at lysine residues within proteins (lysine acetylation or N-ε-acetylation) and at the N-terminus of proteins (Nt-acetylation (NTA) or N-α-acetylation). Protein NTA is among the most common modifications of eukaryotic proteins and is carried out by N-terminal acetyltransferases (NATs)^1–3^. Most soluble yeast (>50%) and human (80%) proteins are Nt-acetylated. NATs catalyze the transfer of an acetyl moiety from acetyl coenzyme A to the α-amino group of the nascent polypeptides as they emerge from the ribosome. This is analogous to the transfer of an acetyl moiety to the lysine side chain by histone acetyltransferases^2^. In the case of the NATs, each NAT type, NatA–NatF, is composed of one or more specific subunits and acetylates defined subsets of substrates based on the identity of the very first amino acid residues^3–11^. The NatA complex (NAA10 and auxiliary Naa15 plus other potential proteins) is the major acetyltransferase with 40–50% of all human proteins being potential substrates. *NAA10* encodes NAA10, the catalytic subunit of NatA, co-translationally N-acetylating proteins with Ser, Ala, Thr, Gly, and Val N-termini.

The first human genetic disease directly coupled to this pathway was named Ogden syndrome (OS), in honor of the hometown Ogden, Utah from which Family 1 originates^3,12,13^. In this study, a total of five deceased boys in a family in Utah and three deceased boys in a family in California were revealed to possess a mutated *NAA10* gene (with missense change p.S37P). These boys had a distinct combination of an aged appearance, craniofacial anomalies, hypotonia, global developmental impairments, structural heart defects at birth, cardiac arrhythmias, and cardiomegaly. Structural modeling and molecular dynamics simulations of the human NatA and the OS p.Ser37Pro mutant highlighted differences in regions involved in catalysis and at the interface between NAA10 and the auxiliary subunit Naa15^14^. Biochemical data further demonstrated a reduced catalytic capacity and an impaired interaction between the NatA subunits NAA10 p.Ser37Pro and Naa15, in addition to a reduced interaction between NatA p.S37P and Naa50 (NatE)^14^. Finally, NTA analyses revealed a mild (>10%) decrease in NTA for only 37 NatA and NatE substrates in OS cells, which suggests that the effect of this variant is not on a global proteome-wide level but is rather very likely focused on only a few substrates in specific tissues^14^.

Despite its ubiquitous nature, NTA is an incredibly understudied protein modification, with very few reports on the *mammalian* N-terminome. In the past few years, the first instance of NTA relevance to cardiac function was reported, involving NTA of the cardiac voltage-gated sodium channel, Nav1.5, in tissues from end-stage heart failure patients^15,16^. Indeed, protein quality control is of major relevance in heart failure^17^. Also, a 2015 study linked NTA and N-end rule degradation to blood pressure regulation^18,19^. N-terminal mutants of Rgs2, a key G-protein regulator, are differentially processed by NATs and the two branches of the N-end rule pathway, leading to an imbalance in the signaling governing blood pressure. This was the first demonstration of NTA-prompted degradation of proteins in mammalian cells. In addition, variants involving NTA are now being found in other diseases. A recent study showed that de novo variants in *NAA15* are involved in congenital heart defects^20^, consistent with the cardiac anomalies seen in OS. Another study identified a missense variant in *NAA10* in a proband with intellectual disability^21^. Further, a *NAA10* splice-site variant resulting in apparent knockdown of full-length *NAA10* expression alongside minor expression of a truncated NAA10 was found in one family with Lenz microphthalmia syndrome (LMS)^22^. Additional families with missense variants in *NAA10* are now being reported more frequently, some of which will be discussed further below. Thus far, there is no reported case of a human hemizygous male or homozygous female with truncating variants in *NAA10*, so it is perhaps possible that a complete knockout in humans might be embryonic lethal. The knockdown phenotypes of human NATs in cell culture suggest that protein NTA is an essential modification in human cells to maintain proliferation and survival, but functional insights and mammalian in vivo models are lacking. One group recently reported embryonic and neonatal lethality in a mouse knockout of *NAA10*, with the suggestion that placental insufficiency might contribute to the embryonic lethality and that hydrocephaly might contribute to postnatal death^23^. Among the features that are present in the *NAA10* knockout mice, some are shared by patients with *NAA10*-related syndrome, including low birth weight, postnatal growth failure, and dilated brain ventricles^23^.

In regard to more common diseases and basic biology, there is emerging evidence that NTA of proteins is overexpressed or otherwise dysregulated in a variety of cancers, including lung, prostate, and liver cancers^24–30^. NTA has been linked to neurodegenerative diseases such as Parkinson’s, Alzheimer’s, and Huntington’s Disease, with NatA/NAA10 having been shown to contribute to the regulation of amyloid β-protein generation, to modulate the stabilization of Sup35 amyloid formation, and to prevent aggregation of Htt^31–36^, supporting the importance of NTA in the progression of these diseases. Current findings link NTA to degradation of some proteins, via Ac/N-degron-mediated recruitment of specific ubiquitin ligases^7,8,19,37,38^. NTA may also influence protein complex formation, as exemplified by the NEDD8 ligation enzymes^39^, along with prion formation^32^. Also, protein-specific targeting to membranes of the nucleus^40^, Golgi^41,42^, and lysosomes^43^ was shown to require NTA but a general role in targeting is not supported^44^.

Below, we will present the steps and workup needed for establishing a diagnosis, then a clinical description and differential diagnosis for *NAA10*-related syndrome, and then finally a molecular description related to the mechanisms for these disorders.

### Establishing the diagnosis

The diagnosis of *NAA10*-related syndrome is established in a male proband with the identification of a disease-contributory hemizygous *NAA10* variant and in a female proband with the identification of a disease-contributory heterozygous *NAA10* variant by molecular genetic testing (see Tables 1, 2, 3).

**Table 1:** *NAA10*-related syndrome: genes and databases

Gene	Chromosome locus	Protein	Locus specific	HGMD
NAA10	Xq28	N-alpha-acetyltransferase 10	http://databases.lovd.nl/shared/genes/NAA10	NAA10

Data are compiled from the following standard references: Gene symbol from HGNC; chromosomal locus, locus name, critical region, complementation group from OMIM; protein name from UniProt

**Table 2:** OMIM entries for *NAA10*-related syndrome

300013	N-ALPHA-ACETYLTRANSFERASE 10, NatA CATALYTIC SUBUNIT; NAA10
300855	OGDEN SYNDROME; OGDNS
309800	MICROPHTHALMIA, SYNDROMIC 1; MCOPS1

**Table 3:** *NAA10* variants reported to date

DNA nucleotide change	Predicted protein change	Number of individuals Identified with verified variant	Reference sequences
c.109T>C	p.Ser37Pro	8 males, 5 asymptomatic maternal carriers	NM_003491.3 NP_003482.1
c.128A>C	p.Tyr43Ser	2 males, 1 affected maternal carrier	
c.247C>T	p.Arg83Cys	8 females, 2 males	
c.319G>T	p.Val107Phe	1 female	
c.346C>T	p.Arg116Trp	1 male, 1 female	
c.382T>A	p.Phe128Ile	2 females	
c.384T>A	p.Phe128Leu	1 female	
c.471+2T>A^a^	Truncated protein	4 males, 3 affected maternal carriers	

Note on nomenclature: This follows the standard naming conventions of the Human Genome Variation Society (http://www.hgvs.org)

^a^This variant is found in one family with LMS and is discussed separately as a Genetically Related (Allelic) Disorder

Molecular genetic testing approaches can include a combination of gene-targeted testing (single-gene testing or a multi-gene panel) and genomic testing (comprehensive genomic sequencing) depending on the phenotype.

Gene-targeted testing requires that the clinician determine which gene(s) are likely involved, whereas genomic testing does not. Because the phenotype of *NAA10*-related syndrome is broad, individuals with the distinctive findings described below are likely to be diagnosed using gene-targeted testing (see Option 1), whereas those with a phenotype indistinguishable from many other inherited disorders with intellectual disability are more likely to be diagnosed using genomic testing (see Option 2).

### Option 1

When the phenotypic findings suggest the diagnosis of *NAA10*-related syndrome, molecular genetic testing approaches can include single-gene testing or use of a multi-gene panel.

Single-gene testing: Sequence analysis of *NAA10* is performed first. If no disease-contributory variant is found, gene-targeted deletion/duplication analysis could be considered; however, to date no exonic or whole gene deletions have been reported.

A multi-gene panel that includes *NAA10* and other genes of interest (see Differential Diagnosis) may also be considered. Note: (1) The genes included in the panel and the diagnostic sensitivity of the testing used for each gene vary by laboratory and over time. (2) Some multi-gene panels may include genes not associated with the condition discussed herein; thus clinicians need to determine which multi-gene panel provides the best opportunity to identify the genetic cause of condition at the most reasonable cost while limiting secondary findings. Of note, given the rarity of *NAA10*-related syndrome, many panels for intellectual disability may not include this gene. (3) Methods used in a panel may include sequence analysis, deletion/duplication analysis, and/or other non-sequencing-based tests.

### Option 2

When the phenotype is indistinguishable from many other inherited disorders with intellectual disability, molecular genetic testing approaches can include genomic testing (comprehensive genomic sequencing; recommended) and/or gene-targeted testing (multi-gene panel; to consider).

Comprehensive genomic sequencing (when clinically available) includes exome sequencing and genome sequencing.

OR

A multi-gene panel that includes *NAA10* and other genes of interest (see 'Differential diagnosis' section) may be considered; however, given the rarity of *NAA10*-related disorder many panels for intellectual disability may not include this gene.

### Clinical description

#### Hemizygous males

Males with an *NAA10-*related syndrome present at different ages; those with arrhythmias may present prenatally; while others are apparent at birth with cardiac concerns, hypotonia, and dysmorphic features; and others come to attention later with relatively nonspecific developmental/intellectual and growth impairments. There are a number of findings in a variety of body systems that have been reported in two or more individuals with this disorder. It is noted that there is a wide spectrum among individuals with this disorder with regard to number of findings and severity of phenotype (see Tables 1, 2, 3).

Prenatal presentation. There may be fetal arrhythmias detected (see 'Cardiac manifestations' below). Prenatal growth is in the normal to low normal range.

Cardiac manifestations include cardiac arrhythmias and/or congenital heart defects.

- Cardiac arrhythmias can present in many forms, including premature atrial contractions, premature ventricular contractions, supra-ventricular tachycardia, ventricular tachycardia, atrial fibrillation, ventricular fibrillation, bradycardia, intraventricular conduction impairments, and *torsade de Points*. Other electrocardiogram (ECG) abnormalities may include PR prolongation, repolarization delay, QT prolongation, and nonspecific T-wave abnormalities. The age of onset for these symptoms can vary from prenatal course to adulthood. Cardiac arrhythmias can occur in the absence of structural defects.
- Congenital heart defects can include patent ductus arteriosus, patent foramen ovale, ventricular septal defect, atrial septal defect, pulmonary artery stenosis, and bicuspid aortic valve.

#### Neonatal-onset hypotonia and other central nervous system concerns

- Neonatal-onset hypotonia is common. It may be associated with:
  - poor feeding
  - dysphagia
  - apneic episodes
  - apparent life-threatening events related to aspiration
- Brain abnormalities revealed by imaging studies:
  - enlarged ventricles
  - cerebral dysgenesis
  - nonspecific findings may include prominence of the Sylvian fissure, immature myelination of the splenium

Dysmorphic features: This may include large fontanels, wrinkled forehead, hypertelorism, prominent eyes, downslanted palpebral fissures, thick eyelids, ears appearing large compared to body size, flared or prominent nostrils, short columella, protruding upper lip or long philtrum, micrognathia or retrognathia, narrow palate, and small hands and feet.

Developmental impairments becomes apparent with

- Delayed motor milestones
- Delayed speech
- Learning difficulties in school
  - Intellectual disability varies from mild to severe
  - Behavioral disorder(s) including hyperactivity, short attention span, attention deficit hyperactivity disorder (ADHD), aggressivity, unmotivated laughter, hand biting, stereotypic behaviors, self-hugging, compulsive and obsessive behaviors, and autistic features.

Postnatal growth restriction is present in most individuals affecting height, weight, and head circumference. This is typically noted within the first year of life.

Skeletal findings can include late closing fontanels, short neck, scoliosis, pectus excavatum, clinodactyly, hip dysplasia, metatarsus valgus, and broad or relatively large halluces.

Skin and other ectodermal findings can include redundancy or laxity of the skin with minimal subcutaneous fat, cutaneous capillary malformations, and recurrent eczema. Scalp hair and eyebrows are often very fine in early years of life.

Genitourinary. More than half of affected males have inguinal hernia, cryptorchidism, and small testes. Glomerulosclerosis and cystic renal dysplasia have also been reported.

Recurrent infections are common and can include frequent otitis media, multiple gastrointestinal and respiratory viral infections, and post-operative infection. There is no known specific immune deficiency.

Other rare findings that have been reported include:

- Umbilical hernia

#### Heterozygous females

The phenotype of a female heterozygous for an *NAA10* disease-contributory variant can range from asymptomatic to having some of the same clinical findings as affected males, including a range of intellectual disability and cardiac findings depending on specific variants and presumed favorable vs. non-favorable X-chromosome inactivation (see Tables 1, 2, 3). In addition, X-chromosome skewing can occur in different ratios in different tissues, particularly if expression of the protein from the mutated X-chromosome confers either a growth advantage or disadvantage in specific tissues.

Testing of the asymptomatic mothers of the males with OS identified nearly complete skewing toward the wild-type allele^14^. These female carriers in the OS family in Utah never underwent intellectual testing (intelligence quotient (IQ), so it is not known if they have any small degree of intellectual disability. However, all of them were able to live independently and have children, so they either had completely normal intellectual functioning or perhaps only mild intellectual disability, if at all. Skewed X-chromosome inactivation of B cells in female carriers of different *NAA10* disease-associated variants has been observed^45^, but the direction of skewing was not demonstrated, making it very difficult to speculate about the impact of X-chromosome skewing on the phenotypic expression in those individuals.

In addition, if skewed X-chromosome inactivation occurs in tissues other than that tested (typically blood), it may not be detected in females who have a heterozygous *NAA10* disease-contributory variant. The below summarizes some of the findings to date:

- Two girls were reported with severe global development impairments and intellectual disability in whom skewing of X-chromosome inactivation was not observed^46,47^. One of the girls was reported to also have anomalies including pulmonary artery stenosis, atrial septal defect, prolonged QT interval, broad great toes, mild pectus carinatum, truncal hypotonia, hypertonia of extremities, and stereotypic behaviors^46^.
- Casey et al. (2015) reported two affected male siblings with a maternally inherited variant, with the mother having learning disability (but no formal IQ testing reported), a prolonged QT interval, and premature coronary artery disease^48^. X- chromosome inactivation studies showed a normal non-skewed (random) X-chromosome inactivation pattern in the mother’s blood.
- Saunier et al. (2016) reported 10 female probands with severe global development impairments and intellectual disability^45^. Commonly shared features include microcephaly, large fontanels, growth failure, hypotonia/hypertonia, behavioral anomalies (hyperactivity, little eye contact, stereotypies, attention deficit, and aggressivity), and cardiac anomalies (septal defects, incomplete right bundle branch block, and long QT). X-chromosome inactivation was random in the majority of tested females (4/5), mildly skewed in the fifth, and only one girl had 100% skewed X-inactivation.

The major point is that future studies should determine the direction of X-chromosome skewing and use formal IQ testing to determine any degree of intellectual disability in females carriers, preferably compared to female non-carrier siblings.

### Genotype–phenotype correlations

Different variants in *NAA10* lead to various degrees of impairment in the enzymatic activity of NatA; however, the phenotypic severity does not seem to correlate with the remaining levels of the enzyme, at least not as measured using in vitro assays. It is also worth noting that the in vitro activity was measured with an MBP-hNAA10 fusion protein alone and not in the context of the NatA complex containing NAA10 and Naa15. Obviously, this is also far removed from the complex situation in vivo both during development and throughout the life of an organism. Nevertheless, it is defined here that all patients with *NAA10*-related syndrome should present with developmental impairments, intellectual disability, and postnatal growth failure to some degree, with some patients also having cardiac and skeletal anomalies. In general, male patients with *NAA10*-related syndrome have a much more severe phenotype than females, including lethality in infancy, and it is suggested below that at least males with NAA10 p.Ser37Pro should be designated as having OS.

- Eight males with NAA10 p.Ser37Pro were the earliest reported individuals with mutations in *NAA10*, designated at the time as having OS^12,13^ but now part of the larger *NAA10*-related syndrome, and also among the most severely affected ones. The variant itself was shown to only have a mild reduction for amino-terminal acetylation of only a few NatA substrates in patients’ cells^14^, in part due to a less efficient binding between NAA10 and its auxiliary partner Naa15^14^. Patients with OS are characterized by a distinct combination of dysmorphic features, cardiac dysfunction, hypotonia, global developmental impairments, and growth failure. Cardiac dysfunction may include congenital heart defects and cardiac arrhythmias, including QT prolongation and other ECG abnormalities. The presenting proband (Family 1, Individual III-4) had QTc intervals ranging from 403 to 479 ms while alive. The average life span of OS patients was 10.2 months, with the maximum age of death at 16 months; however, in the absence of intensive medical intervention, the last boy born with OS (Individual III-6 in Family 1) only lived 4.5 months. The cause of death in all these patients seems most likely to have been related to underlying cardiac arrhythmias. In addition to this, all of the affected children in both families with OS were noted to have large and, in some cases, persistently open fontanels. For one child (Family 1, Individual II-1), computed tomographic scanning revealed enlarged ventricles. For another child (Family 1, Individual III-4, at 15 months of age), it was reported that magnetic resonance imaging showed “moderate lateral and third ventricular dilatation without identified cause,” but a pathologist reported in a somewhat later autopsy of Family 1, Individual III-4 at 15 months of age that “the ventricles were uncompressed and undilated.” A different autopsy report on Family 1, Individual III-7, at 5 months and 3 weeks of age, revealed that the lateral ventricles were “slightly dilated.” All of the children had respiratory depression and apneic episodes, along with varying course of hypotonia and/or hypertonia (including documented hyperreflexia in at least one case (Family 2, Individual III-2))^12^. The mothers of the S37P males were never diagnosed with intellectual disability or any other serious medical condition. It was previously shown that their X-chromosome inactivation was nearly completely skewed toward the wild-type allele in blood, and it is possible that this might have been true in other tissues of their bodies as well. Much more detailed clinical descriptions of patients with OS have been presented in detail elsewhere^12,49^.
- Two male siblings with a maternally inherited hemizygous NAA10 p.Tyr43Ser variant have milder clinical phenotype than OS patients, featuring intellectual disability (based on their functioning status, as IQ testing was not reported), facial dysmorphism, scoliosis, and long QTc (ranges reported from 494 to 533 ms^48^. Patients’ cardiac events start at a much later age, usually around young adulthood. The above children’s mother, with heterozygous NAA10 p.Tyr43Ser, has a learning disability, a prolonged QTc interval (observed between 460 and 500 ms), and premature coronary artery disease. X-chromosome inactivation studies showed a normal X-chromosome inactivation pattern in blood. In vitro assays showed a significant decrease in catalytic activity of the NAA10 p.Tyr43Ser mutant enzyme and a reduced stability of NAA10 p.Tyr43Ser compared to the wild-type.
- Two male siblings with *NAA10* p.Arg83Cys variant were reported. One had persistent pulmonary hypertension, supraventricular tachycardia, mild ventricular hypertrophy, generalized hypotonia, and ventriculomegaly in the brain. He lacked spontaneous respirations and died at age 1 week. Later, another male fetus in this family was conceived but the pregnancy was terminated due to the same variant being identified prenatally. Eight females with *NAA10* p.Arg83Cys variant have now been reported, with one being the sibling of the above two males with *NAA10* p.Arg83Cys variant^45,50^. All girls show moderate-to-severe developmental impairments, small hands and feet are found in some, and hypotonia and behavioral issues (autistic-like behaviors) are common. Cardiac manifestation in these girls are rather mild, with one having some mild birth defects, one with incomplete right bundle branch block, one with long QT (max QTc 484 ms), and one with left ventricular hypertrophy subsequent to systemic hypertension. Findings of brain studies include electroencephalogram (EEG) abnormalities, hippocampal dysgenesis, white matter volume loss, a thin corpus callosum, ventriculomegaly, and hypoxic–ischemic encephalopathy (HID). One of the female patients with an p.Arg83Cys change in *NAA10* was reported to have supernumerary vertebrae, although no further details were provided^45^. Four other females carrying variants in *NAA10* were reported as having either pectus carinatum or excavatum. Ventriculomegaly was reported in several female probands with missense variants in *NAA10*, and one of these female patients with an p.Arg83Cys missense in NAA10 (#9 in Table 1 of that paper) was reported as having intraventricular hemorrhage (IVH) in the occipital horn, HID, and a ventriculo-peritoneal (VP) shunt^45^. It is possible that this sequence of events is compatible with hydrocephaly with clinical signs and symptoms that required the placement of the VP shunt, given that the IVH may have caused the hydrocephaly. Another p.Arg83Cys female patient (now 14 years of age) was recently reported to also have epileptic encephalopathy, progressive white matter volume loss, progressive hypersomnolence, and extrapyramidal features responding to carbidopa-levodopa^50^. The unique features in this girl suggest a progressive or evolving neurological condition, which may need to be differentially diagnosed from other conditions such as hypomyelinating disorders. The long-term follow-up study of this particular female patient (so far the oldest living patient with an *NAA10* variant) provides unique knowledge of the trajectories and prognosis of this condition as well as some of the necessary monitoring parameters in such patients. The variant of NAA10 p.Arg83Cys was reported to not affect the stability of NAA10, but it caused a clear reduction (~60%) of the catalytic activity of the MBP-NAA10 fusion protein in vitro^45^.
- A girl with NAA10 p.Val107Phe was reported to have severe developmental impairments, short stature with postnatal growth failure, microcephaly, structural heart defects, and QT prolongation identified at a young age (4 year and 8 months)^46^. She also has behavioral anomalies, such as little eye contact, self-hugging, and repetitive hand movements. In vitro studies showed a nearly abolished enzymatic activity of the MBP-NAA10 fusion protein in vitro.
- A boy with NAA10 p.Arg116Trp was reported to have severe intellectual disability (based on formal IQ testing) and developmental impairments^46^. He also had small hands/feet, generalized epileptiform activity on EEG, hyperactivity, autistic behaviors, and postnatal growth failure. Enlarged ventricles were found on brain imaging. A female was also reported with NAA10 p.Arg116Trp variant, who presented with mild features including moderate developmental impairments, mild intellectual disability, hypotonia, dilated lateral ventricles, developmental coordination disorder, and ADHD^45^. Her heart examination was almost normal, with only incomplete right bundle branch block. In vitro studies showed only a very mild reduction in the catalytic activity of the MBP-NAA10 fusion protein in vitro^45^.
- One girl with NAA10 p.Phe128Ile was reported sharing several features with the girl with NAA10 p.Val107Phe, including developmental impairments, feeding difficulties, postnatal microcephaly, axial hypotonia, and peripheral hypertonia. The X-chromosome inactivation pattern in her blood cells was random. Furthermore, a different variant that caused amino acid substitution at the same position, NAA10 p.Phe128Leu, was found in two unrelated females; the phenotype of these girls included severe developmental impairments, feeding difficulties, abnormal EEG findings, parenchymal atrophy, prenatal and perinatal ventriculomegaly, mild left ventricle diastolic dysfunction, cortical vision impairment, and behavior issues (stereotypies, hand washing, uncertain eye contact). Both NAA10 p.Phe128Leu and NAA10 p.Phe128Ile were shown to reduce the stability of NAA10 and also lead to a near loss (>90%) of the MBP-NAA10 fusion protein activity in vitro.

### Nomenclature

Early reports were focused on the individuals who first came to medical attention and who were the most severely affected individuals and reported as having OS, with the first family living in Ogden, Utah^12^. Use of genomic testing for individuals with phenotypes that were difficult to diagnose on clinical grounds alone (e.g., nonspecific intellectual disability) enlarged the spectrum of clinical findings associated with disease-contributory variants in *NAA10*. Therefore, *NAA10-*related syndrome is the preferred name for the overall heterogeneous presentation with mutations in *NAA10*. It is suggested that OS should be reserved for male infants with the fatal neonatal presentation.

### Prevalence

To date, at least 26 individuals of European descent have been reported to have *NAA10-*related syndrome, not including one variant leading to reduced expression of a truncated form of NAA10 (as described below)^22^. The overall worldwide prevalence is unknown. Data are also not available for estimating the prevalence based on ethnic background.

### Genetically related (allelic) disorders

- LMS has also been associated with a disease-contributory variant in NAA10 in one family with at least four affected brothers^22^. This involves a variant in the intron 7 splice donor site of NAA10. This variant co-segregated with the phenotype and cDNA analysis showed aberrant transcripts. Patient fibroblasts lacked expression of full-length NAA10 and displayed cell proliferation defects. Although the affected individuals in this family do have developmental impairments, intellectual disability, and some postnatal growth failure, thus meeting the criteria for NAA10-related syndrome, LMS is characterized by unilateral or bilateral microphthalmia and/or clinical anophthalmia with malformations of the ears, teeth, fingers, skeleton, and/or genitourinary system. In this family, female carriers of the NAA10 disease-contributory variant had two to three toe cutaneous syndactyly and short terminal phalanges in the hand without other findings. Given that the ocular phenotype is so distinct, this is currently being listed here as a genetically related (allelic) disorder. It is worth noting that the proband in the original family for LMS was reported to die at age 28 years with complications of uremia, with autopsy revealing bicuspid aortic valve with mild coarctation of the aorta; aplasia of the left kidney, ureter, and renal vessels; hydronephrosis of the right kidney with parenchymal atrophy; cryptorchidism; and spina bifida occulta^51^. We are not aware if any DNA is available still for this proband, and we cannot find anything in the literature concerning confirmed variants for this original case. There is one other report in which a proband with presumed Lenz syndrome had a maternal aunt with poor vision and ureteral obstruction since childhood, and her child (the proband’s maternal half-brother) had short stature and dysplastic kidneys associated with a left obstructive nephropathy secondary to stenosis of the left ureter^52^. Unfortunately, this family declined DNA linkage analysis, so the genetic variant(s) involved are unknown; however, we remark on these families here primarily to highlight the cardiac and urogenital defects that did reveal themselves over time with this disorder, which does overlap with some of the defects that have been noted in other patients with NAA10-related syndromes^12^^,^^47^^,^^50^. It is worth noting that 9/13 (69%) of the girls reported with missense variants in NAA10 had some milder form of eye or visual anomalies, including astigmatism, cortical visual impairment, hyperopia, and/or myopia^47^.

### Differential diagnosis

Phenotypic features associated with *NAA10* disease-contributory variants, particularly including intellectual disability, may not be sufficient to diagnose an *NAA10*-related disorder. Therefore, all genes known to be associated with intellectual disability (over 130 have been identified, see OMIM Phenotypic Series) should be included in the differential diagnosis of *NAA10*-related syndrome. A few other disorders to consider as a possible diagnosis are listed below.

#### Timothy syndrome (Long QT syndrome 8)

Timothy syndrome is a multisystem disorder characterized by cardiac (prolonged QT interval, functional 2:1 atrioventricular (AV) block with bradycardia, tachyarrhythmias, and congenital heart defects), hand/foot, facial, and neurodevelopmental features. Shared features include prenatal onset of cardiac rhythm disturbance, dysmorphic facial features, hypotonia, and developmental impairments. However, the heart rhythm issue in Timothy syndrome usually includes 2:1 AV block, and individuals commonly have cutaneous syndactyly. Timothy syndrome is caused by a heterozygous disease-contributory variant (typically de novo) in *CACNA1C*, the gene encoding the calcium channel Cav1.2 α subunit.

#### Hutchinson–Gilford progeria syndrome (HGPS)

Infants with *NAA10*-related syndrome may have an aged appearance, which raises a suspicion of a progeria-liked syndrome. Children with HGPS usually have normal fetal and early postnatal development and normal cardiac findings until ages 5–8 years or later^53^. In contrast, the cardiac phenotype in *NAA10*-related syndrome may be evident in early infancy, and autopsy examination of infants with *NAA10*-related syndrome reveals that they did not have the characteristic premature arteriosclerosis or degeneration of vascular smooth muscle cells observed in HGPS.

#### Sudden infant death syndrome (SIDS)

SIDS is the unexplained sudden death of an otherwise healthy child younger than 1 year of age, usually during sleep. Although the pathophysiological mechanism underlying SIDS is unknown, it is widely believed that the death involves immature cardiorespiratory control in conjunction with a failure of arousal from sleep^54^. However, infants with *NAA10*-related syndrome often present congenital anomalies and/or developmental impairments, and premature death has been related to arrhythmia-induced cardiogenic shock.

### Molecular genetics and mechanisms

#### Normal gene product

*NAA10* encodes N-alpha-acetyltransferase 10 (NAA10), the catalytic subunit of the major amino-terminal acetyltransferase A complex (NatA). NatA transfers the acetyl moiety from acetyl-coenzyme A (Ac-CoA) to the primary α-amino group of a nascent polypeptide as it emerges from ribosomes. Nα-terminal acetylation (NTA) is among the most common protein modifications in eukaryotes^3^, but the biological implications of this protein modification are relatively understudied. NAA10 (isoform 1, encoded by transcript NM_003491.3) has 235 amino acid residues and a molecular weight of 26 kDa. NAA10 contains a Gcn5-related N-acetyltransferase domain, as well as an Ac-CoA-binding domain, so it is thought that its main function relates to NTA, although other functions have also been proposed^3^. Alternate splicing results in multiple *NAA10* transcript variants, the longest of which is NM_003491.3 with eight exons. *NAA10* has a universal expression pattern in human fetal and adult tissues, mainly located in the cytoplasm. Out of ~63,000 humans that were sequenced as part of the Exome Aggregation Consortium^55^, there are only two stop-gain variants in *NAA10*. Both are heterozygous in females, p.Arg158Ter and p.Ser233Ter, with the former variant well outside of the enzymatic domain and the latter at the very C-terminus of the protein, which suggests that complete knockout of this protein in males might be embryonic or neonatal lethal. However, this is not entirely consistent with the splice-site variant giving a fairly dramatic knockdown (or perhaps even complete knockout) of *NAA10*, yet the males in the one family with LMS grow to adulthood with a severe ocular malformation phenotype^22^. However, the paper does show via western blotting from one patient sample that a small amount of truncated protein product is possibly expressed, which could be acting to rescue the phenotype and/or acting in a dominant-negative manner. It is also possible that a very small amount of full-length *NAA10* (not detected by reverse transcriptase–polymerase chain reaction and western blotting reported in the publication) is expressed and that this is sufficient to rescue these patients from embryonic or neonatal lethality^22^. As more and more “normal” humans are sequenced as part of population cohort studies, it will be interesting to see if any putative loss-of-function alleles in *NAA10* are ever found in any human males.

#### Abnormal gene product

Functional studies demonstrate that NAA10 p.Ser37 is located in the globular region of NAA10 that interacts with Naa15, the auxiliary subunit of the NatA enzyme; both are essential for NatA activity. Variants in *NAA15* have also been found in patients with congenital heart disease, intellectual disability, and/or autism^20,56,57^. Perturbations of the NAA10**–**NAA15 interaction interface and changes in the flexibility of key regions for substrate binding can explain the impaired NAT activity and complex stability of NatA^14^. The disease-associated p.Ser37Pro variant has decreased enzymatic activity when tested with in vitro acetylation assays^12,14^. Similarly, quantitative analysis of proteome-wide acetylation in B-cells and fibroblasts of affected individuals revealed a very mild reduction of the in vivo acetylation status of a very small number of proteins^14^, which provides further support to the idea that the phenotypes are likely related to the decreased acetylation of only a small number of (currently unknown) proteins both spatially and temporally during development.

The functional testing for other *NAA10* disease-contributory variants has been based on testing in vitro purified NAA10 and/or expression in human cell lines. For example, NAA10 p.Tyr43Ser showed a significantly decreased catalytic activity and reduced stability compared to wild-type NAA10^48^; NAA10 p.Arg116Trp showed high remaining catalytic activity, consistent with the relatively mild clinical presentation^46^; the de novo p.Val107Phe identified in a girl had almost abolished catalytic activity, consistent with more severe clinical features^46^. However, it is important to note that phenotypic severity does not always correlate with the levels of in vitro catalytic activity.

In addition, one research group recently reported more in vitro studies on some of the above-mentioned NAA10 variants including p.S37P, p.V107F, and p.R116W in regulating global DNA methylation and genomic imprinting^23^, which provide a new idea regarding the mechanism of *NAA10*-related syndrome, in addition to or instead of a dysregulated protein acetylation pathway. This study suggested that these pathogenic *NAA10* mutations disrupt binding of NAA10, through a putative homeobox DNA binding motif, to imprinting control regions/differentially methylated regions of mouse *H19*, which further affects the recruitment of DNMT1 (DNA methyltransferase 1), giving rise to a defective DNA methylation and imprinting pattern of genes that are important for development. The existence of a homeobox DNA binding motif in NAA10 has not been proven definitively; however, if the observations in this paper are replicated in the future, then the varying effects on DNA methylation in different NAA10-regulated imprinted genes may help explain the stochastic nature of the phenotypic effects in *NAA10*-KO mice as well as in individuals with *NAA10*-related syndrome.

## Summary and conclusion

The original two families with NAA10 p.Ser37Pro had only males with a relatively severe neonatal lethal phenotype, which was named OS in honor of the hometown (Ogden, Utah) where the first family resided. This variant segregated in these pedigrees in multiple affected individuals, and the maternal carriers did not have any documented intellectual disability (but there was never any formal intelligence testing). Since that initial description, others have reported different variants in *NAA10*, sometimes with only a mild intellectual disability phenotype in females. The recurrence of some of these variants in multiple affected individuals increases the chances that these variants are indeed contributing to these phenotypes. The future identification of other variants will help to confirm or refute the contribution of any variants that are currently reported in only a single family or even in just a single person. Although developmental impairments/intellectual disability may be the only presenting feature, some of these individuals have additional cardiac, growth, and dysmorphic findings that vary in type and severity, reflecting the extensive phenotypic variability that seems be linked to the various functions of *NAA10*. For the one family with LMS with a splice-site variant in *NAA10*, in which patient-derived fibroblasts lacked the expression of full-length NAA10 and displayed a cell proliferation defect, it is unknown why this family alone has such a dramatic ocular phenotype in males. Therefore, given the substantial phenotypic variability, we are recommending that this disorder should be referred to more broadly as *NAA10*-related syndrome.
